# Characterization and Optimization of Isotachophoresis Parameters for Pacific Blue Succinimidyl Ester Dye on a PDMS Microfluidic Chip

**DOI:** 10.3390/mi11110951

**Published:** 2020-10-22

**Authors:** Himali Somaweera, Zachary Estlack, Jasmine Pramila Devadhasan, Jungtae Kim, Jungkyu Kim

**Affiliations:** 1 Department of Mechanical Engineering, Texas Tech University, Lubbock, TX 79409, USA; himashcool@gmail.com (H.S.); jasminepramila.d@gmail.com (J.P.D.); 2 Department of Mechanical Engineering, University of Utah, Salt Lake City, UT 84112, USA; zachary.estlack@utah.edu; 3 KIST-EUROPE, 66123 Saarbrucken, Germany; tais@kist-europe.de

**Keywords:** isotachophoresis, PDMS (polydimethylsiloxane) microfluidic chip, pacific blue succinimidyl ester, preconcentration, lactate dehydrogenase

## Abstract

Isotachophoresis (ITP) for Pacific Blue (PB) dye using a polydimethylsiloxane (PDMS) microfluidic chip is developed and characterized by determining the types and concentrations of electrolytes, the ITP duration, and the electric field density. Among candidate buffers for the trailing electrolyte (TE) and leading electrolyte (LE), 40 mM borate buffer (pH 9) and 200 mM trisaminomethane hydrochloride (Tris-HCl) (pH 8) were selected to obtain the maximum preconcentration and resolution of the PB bands, respectively. With the selected TE and LE buffers, further optimization was performed to determine the electric field (EF) density and the ITP duration. These ITP parameters showed a 20–170,000 preconcentration ratio from initial PB concentrations of 10 nM–100 fM. Further demonstration was implemented to preconcentrate PB-conjugated lactate dehydrogenase (LDH) using the PDMS microfluidic chip. By utilizing the quenching nature of PB-LDH conjugation, we were able to identify concentrations of LDH as low as 10 ng/mL. This simple PDMS microfluidic chip-based ITP for PB preconcentration enables highly sensitive biological and chemical analyses by coupling with various downstream detection systems.

## 1. Introduction

Analyzing low-abundance analytes in small volumes of biological samples is a primary challenge when performing highly sensitive biological and chemical analyses [1]. To overcome this challenge, efficient on-chip preconcentration and separation techniques such as field-amplified sample stacking (FASS) [2], dynamic pH junction [3], and isotachophoresis (ITP) [4] are widely used to improve the sensitivity and separation efficiency of downstream analytical tools. Recently, ITP utilizing a difference in electrophoretic mobilities has gained prominence as a means to achieve a million-fold preconcentration ratio by using fluorescence dyes [5,6,7].

To implement ITP with dye molecules properly in a microfluidic chip, a leading electrolyte (LE) and a trailing electrolyte (TE) should be selected along with electric field and duration [8]. A measurement point should also be selected to allow for accurate quantification of the preconcentration amount. Even though these ITP parameters for FITC and Alexa Fluor are well studied, each dye molecule should be characterized to optimize ITP preconcentration. Pacific Blue (PB) succinimidyl ester is an excellent fluorescent probe that is able to conjugate with primary amines by forming a stable carbodiimide bond [9]. The spectral and chemical properties of PB, such as its low pKa, high photostability, and high quantum yield, make PB a superior fluorescent dye and can provide a 200-fold increase in sensitivity with better resolution than fluorescamine derivatization [10,11]. In previous studies, PB has only been characterized for capillary zone electrophoresis (CZE) to detect amino acids with high sensitivity [10]. To preconcentrate PB using ITP, the selection of the TE and LE buffers with other ITP parameters are critical steps. 

In addition, most microfluidic chips used for ITP are fabricated using glass and poly(methyl methacrylate) (PMMA) [12,13]. However, glass microfluidic chips require time-consuming and complicated wet-etching microfabrication processes that must be conducted in a cleanroom [14]. Furthermore, PMMA microfluidic chips require conventional prototyping, such as computer numerical controlled (CNC) milling [15,16], laser ablation [17], and micro-imprinting techniques [18]. In contrast, polydimethylsiloxane (PDMS) is commonly used to fabricate various microfluidic devices because of its low cost, ease of fabrication, biocompatibility, air permeability, and optical clarity [19,20]. Although there are some previous studies using PDMS microfluidic chips for ITP [21,22,23,24,25], they have not been widely adopted due to the hydrophobicity of PDMS surfaces and the unstable electroosmotic flow (EOF) due to non-crosslinked oligomers [20] though polyvinylpyrrolidone (PVP) has been used to mitigate this [24]. These issues must be resolved to achieve effective ITP preconcentrations in PDMS microfluidic chips. 

In this study, by performing a parametric study, various ITP parameters such as the types and concentration of LE and TE buffers, electric field density, and duration were characterized to maximize the PB preconcentration ratio while maintaining spatial ITP resolution using a PDMS microfluidic chip. By analyzing the intensity and width of the PB bands, the optimum ITP conditions were determined to obtain preconcentration ratios from multiple initial PB concentrations in the PDMS microfluidic chip. Further demonstration was performed to preconcentrate and quantify PB-conjugated lactate dehydrogenase (LDH) using the optimized ITP conditions. This study provides a guideline to the experimental design and optimization of ITP to preconcentrate PB and PB-conjugated biomolecules with proper ITP resolution using a simple PDMS microfluidic chip that can easily be incorporated into a wider detection system.

## 2. Materials and Methods

### 2.1. Fabrication and Preparation of PDMS Microfluidic Chip 

A PDMS microchannel (65 mm in length, 50 μm width, and 25 μm height) was designed and fabricated using a standard soft-lithography technique. First, with a photomask (CADART Inc., Bandon, OR., USA) from the microchannel design, molds were created on a 4-inch silicon wafer using SU-8 2015 (Kayaku Advanced Materials, Westborough, MA, USA) [26,27,28]. Uncured PDMS (Sylgard 184, Dow Corning, Midland, MI, USA) was then poured into the mold and cured at 95 °C for 1 h to replicate the microchannels. A featureless PDMS substrate was fabricated by pouring uncured PDMS into a petri dish and cured under the same conditions. After punching inlet and outlet holes, these two PDMS replicas were bonded using oxygen plasma activation. Finally, the PDMS microchannel device was bonded to a glass slide for structural support during the ITP.

The PDMS microchannel was then modified to improve the surface properties of the PDMS since the hydrophobicity and unstable EOF of PDMS are disadvantageous for ITP. The oxygen plasma activation, as well as an EOF suppressor, help minimize these drawbacks during ITP [20]. For this study, we chose polyvinylpyrrolidone (PVP), which is one of the water-soluble polymers commonly used as the EOF suppressor for ITP [24,29]. A 1% (*w*/*v*) PVP (Sigma Aldrich, St. Louis, MO, USA) was prepared and added to all the electrolyte solutions for ITP experiments. 

### 2.2. Reagent Preparation for ITP

A TE stock solution of 200 mM sodium tetraborate (Na_2_B_4_O_7_: Borate) at pH 9 was prepared by dissolving sodium tetraborate decahydrate and boric acid (Fisher Scientific, Pittsburgh, PA, USA) in DI water. Then, 5 mM HEPES solution at pH 7 was prepared using 1 M HEPES (Invitrogen, Waltham, MA, USA). To select the LE, three LE stock solutions were initially prepared by titration: 1 M sodium acetate (pH 4) (acetic acid and sodium acetate: Sigma Aldrich, St. Louis, MO, USA), 1 M sodium chloride (pH 7) (Sigma Aldrich, St. Louis, MO, USA), and 1 M Tris-HCl (pH 9) (tris-base: Fisher Scientific, Pittsburgh, PA, USA, Hydrochloric acid: Macron Fine Chemicals, Center Valley, PA, USA). Tris-HCL was selected since it has been widely used for LE in ITP preconcentration. Other two LEs (sodium acetate and sodium chloride) were chosen as candidate LEs due to their high electrophoretic mobilities. The desired concentrations were achieved by diluting the stock solutions of the electrolytes and PB succinimidyl ester (Life Technologies, Rockville, MD, USA) to the appropriate levels. A 10 µM stock was prepared using dimethyl sulfoxide (DMSO) (Sigma-Life Science, St. Louis, MO, USA). In addition, both a spacer and EOF suppressor should be selected to preconcentrate PB using ITP effectively. For this study, 100 nM sodium acetate and PVP are used for a spacer and the suppressor for all ITP experiments and are mixed with the TE buffer. 

### 2.3. ITP Protocol

**Figure 1:**
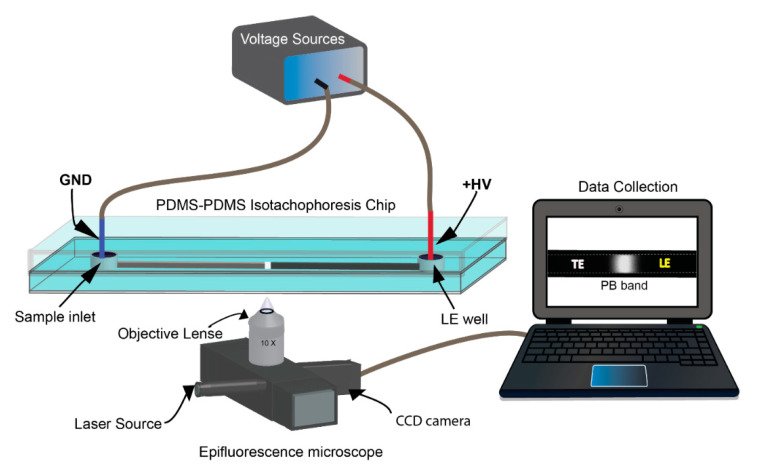
Schematic representation of the experimental setup in which Isotachophoresis (ITP) was used to preconcentrate Pacific blue succinimidyl ester dye (PB) using a simple PDMS microfluidic chip. ITP was performed using a computer-controlled voltage source after filling the sample inlet and outlet with the trailing electrolyte (TE) + PB and the leading electrolyte (LE) under an epi-fluorescence microscope.

First, a PDMS microchannel was filled with DI water and primed by applying an electric field (77 V/cm) for 5 min to stabilize the surface charge on the PDMS surface. The separation channel was then washed three times with DI water and filled with the LE. Second, the sample inlet was rinsed with DI water 2–3 times and then filled with a PB sample prepared in the TE. As illustrated in Figure 1, during the ITP, a high-voltage electrode was inserted into the outlet (LE well), and a ground electrode was placed into the sample inlet (sample well). By using a custom-made high-voltage circuit (EMCO, Allen, TX, USA) with a DC amplifier (HY3003D, MASTECH, San Jose, CA, USA), various electric fields were applied to perform ITP. These procedures were used to determine the optimal electrolytes and electric field for preconcentrating PB using ITP. Between each ITP trial, the microchannel was washed with DI water. During ITP experiments, videos were collected at five different locations downstream from the sample inlet using an automated Ti-E fluorescence microscope (Nikon, Tokyo, Japan; Ti-E with an Andor iXon 3 CCD) and a 10× objective (Nikon, Tokyo, Japan; 0.3 NA) at a 200 ms exposure time. The frames were extracted from the video using a custom-made MATLAB 2019b program and analyzed using ImageJ 1.50.

### 2.4. Determination of the TE and the LE Electrolytes

To select a TE buffer, an initial trial of 5 mM N-2-Hydroxyethylpiperazine-N’-2-Ethanesulfonic (HEPES) at pH 7 and 200 mM borate buffer at pH 9 were used to evaluate ITP performance using 20 mM NaCl (pH 7) as a LE buffer. NaCl was chosen as the LE for the pilot study since it is known to have a high ionic mobility [30] and 123 V/cm was used as the EF and measure the band intensity in the middle of the ITP channel. Once the proper TE type was selected that was able to stack PB effectively, different concentrations of TE were tested as initial trials (from 20 to 100 mM). Then, by using the selected TE, three possible LEs such as sodium acetate (pH 4), sodium chloride (pH 7), and Tris-HCl (pH 8) of 100 mM were evaluated to confirm the stacking effect of Cl^-^ as a leading ion. The pH of all electrolytes was set near their pKa to have the best buffering capacity. After selection of the TE and LE types, the previously mentioned ITP protocol and setup (shown in Figure 1) was used to perform further parametric studies. All experiments in this text were performed in triplicate and all results have less than 5% error unless otherwise noted.

### 2.5. Optimization of Electric Field (EF) and Measurement Point

The focus of this study is to find the optimum EF that can provide a stable ITP environment without electrolysis. Since EF and measurement point are less sensitive to the concentration of LE and TE, once the proper LE and TE were selected, lower-end concentrations (100 mM LE and 40 mM TE) were initially used for EF optimization. Using the ITP protocol mentioned in Section 2.3, the EF optimization was carried out using five different EF densities: 77, 92, 108, 123, and 138 V/cm and measurements occurred at five different points in the channel. All experimental videos were recorded to obtain PB band profiles with respect to time to determine the overall ITP duration.

### 2.6. Optimization of LE and TE Concentrations

Once the optimum EF and measurement point were assigned, the optimum LE and TE concentrations were found. To evaluate the effect of LE and TE concentrations, first, five different concentrations of the selected LE buffer were tested: 100, 140, 175, 200, and 250 mM with the selected TE buffer under an optimal EF as shown in SI. For each LE concentration, the fluorescence intensities, resolutions, separation efficiencies, and widths of the PB bands were measured at the 40 mm point and compared to determine the effect of LE concentration on ITP. The constant measurement location here was used because, regardless of buffer concentration, sample accumulation was poor prior to this point. Figure S1 shows that separation and resolution improve as the measurement location moves further from the sample inlet. In addition, it is seen that distance along the channel increases accumulation, but band length increases as well, ruling out the 50 mm point as well. After evaluating the effect of the LE concentration, three different concentrations of the selected TE (40, 60, and 80 mM borate buffer) were evaluated to investigate the effect of TE concentration on PB preconcentration under optimized EF, measurement point, and LE concentration. 

### 2.7. The Preconcentration Ratio of PB

Once all ITP parameters were determined, a calibration curve (Figure S2) was constructed without ITP by measuring the intensities of known concentrations of PB 10, 25, 100, and 200 nM using a PDMS microfluidic chip. The separation channel was filled with the above concentrations of PB, and the fluorescence intensities were recorded for each concentration. To determine the preconcentration ratio, ITP was performed for five different initial concentrations of PB (10 nM, 1 nM, 100 pM, 10 pM, and 100 fM) under optimized ITP conditions. Linear regression of the calibration curve was used to calculate PB concentrations after ITP. All fluorescent images were background corrected and then analyzed using ImageJ.

### 2.8. LDH Measurement with PB

To demonstrate the preconcentration and separation of a PB-conjugated analyte using ITP, 200 µg/mL of L-LDH from rabbit muscle (Roche Diagnostics) was prepared in DI water. First, a series of 0.01, 0.1, 1, and 100 µg/mL LDH samples were prepared in the optimized TE buffer. Each LDH sample was then mixed with 0.34 µg/mL PB and incubated for 30 min for conjugation. Using the same ITP procedure mentioned above, ITP was then performed to demonstrate LDH preconcentration. In addition, to show the selectivity of this proposed method, preconcentration of LDH with PB was carried out using samples made in a mixture of 1:5 ratio of cell media to TE. The media was used in a HeLa dish culture and removed during conventional media changing. It consisted of Dulbecco’s Modified Eagle Medium, Fetal Bovine Serum, and an antibiotic in a 100:10:1 ratio (Thermofisher Scientific, Waltham, MA, USA).

## 3. Results and Discussion

### 3.1. Selecting Electrolytes to Preconcentrate PB on a PDMS Microfluidic Chip

Using the PDMS microfluidic chip, we selected the proper TE and LE buffers to balance the electrophoretic mobility for PB preconcentration. PB samples were prepared in a TE buffer to obtain concentrated PB plugs in our anionic ITP. Additionally, due to the reactivity of PB with primary amines, the selection of a TE buffer must be non-amine [31]. As the electrophoretic mobility of PB is uncertain, a commonly used non-amine buffer N-2-Hydroxyethylpiperazine-N’-2-Ethanesulfonic (HEPES) [5,32,33] was first evaluated using Cl^−^ (79 × 10^−9^ m^2^ V^−1^s^−1^) as the leading ion. Although the electrophoretic mobility of HEPES at pH 7 (0.7–1 × 10^−8^ m^2^ V^−1^s^−1^) is significantly low compared to Cl^−^, HEPES was unable to provide the proper gradient of electrophoretic mobilities among TE, LE, and PB required to preconcentrate PB. This was unexpected due to the low mobility of HEPES, however parametric experiments rule out both pH and concentration effects. Further work is required to determine the true reason HEPES failed as a buffer for PB preconcentration in ITP. According to previous studies, borate was selected as the second candidate for the TE since borate buffers are commonly used for background electrolytes to analyze anions in capillary electrophoresis (CE), and borate has been used to detect amino acids coupled with PB using CE [10,34]. A borate buffer was tested, and a highly accumulated PB band was obtained. Although HEPES failed to preconcentrate PB experimentally, the electrophoretic mobility range of the borate ion (30 × 10^−9^ m^2^ V^−1^s^−1^) and the Cl^−^ ion was able to stack PB successfully [31,33].

**Figure 2:**
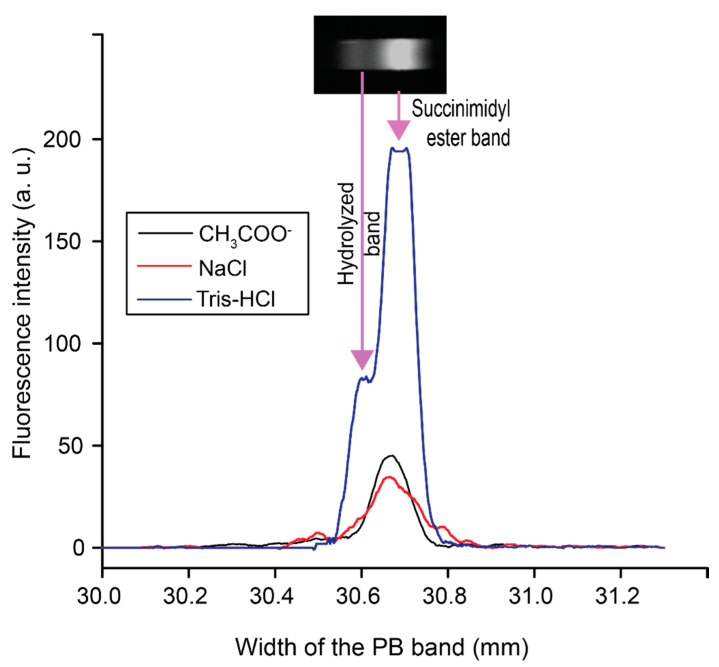
Selection of the leading electrolyte (LE) by evaluating the efficiencies of preconcentration and separation of 100 pM PB. In total, 100 mM of three different types of LE at pH 8 (sodium acetate, sodium chloride, and Tris-HCl) were used to preconcentrate PB, while 40 mM of borate buffer at pH 9 was used as the TE under 123 V/cm. The intensity profiles of the PB band that formed at 30 mm downstream from the sample inlet is shown. The inset image illustrates the formation of two bands of PB due to hydrolysis.

After selecting borate for the TE, we compared the fluorescence intensities of PB with three LE candidates (NaCl, Tris-HCl, and NaCH_3_COO^−^) to confirm the stacking effect with Cl^−^ ion as the leading ion. Figure 2 presents the fluorescence intensity profiles of the PB band under the three different LE buffers. Out of the three different LEs, Tris-HCl showed the highest preconcentration of PB. Another aspect to select LE is to investigate peak resolution. Typically, PB shows two distinct peaks from hydrolysis from capillary electrophoresis [9]. As seen in Figure 2, two PB bands were formed with Tris-HCl at pH 8 as the leading electrolyte along with borate as the trailing electrolyte. The preconcentration under tris-HCL was significant enough that the camera was saturated in the middle of the peak. The remaining ITP parameters for our chip design were optimized and the results are detailed in the supplemental information.

### 3.2. The Preconcentration Ratio of PB

**Figure 3:**
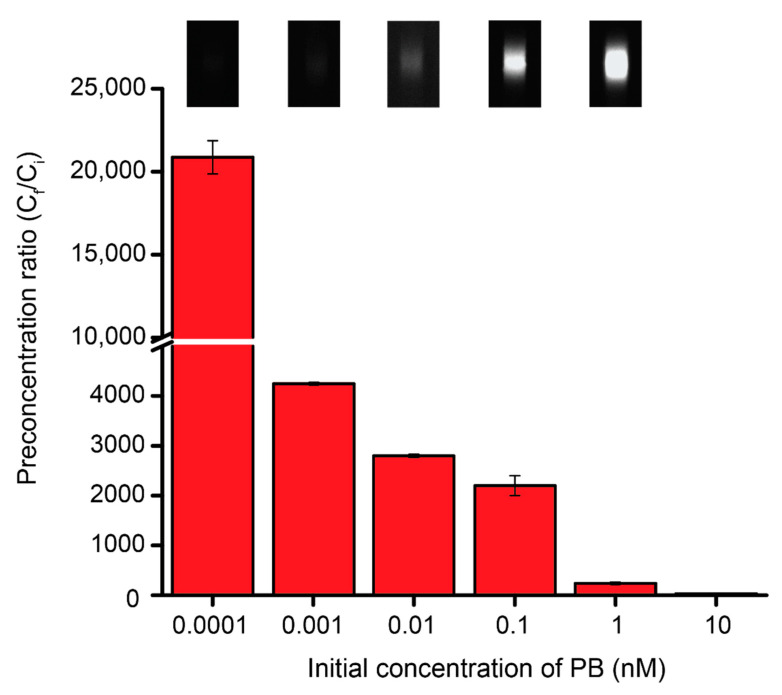
A plot of initial concentration of PB versus preconcentration ratio of different PB concentrations (10, 1, 0.1, 0.01, 0.001, and 0.0001 nM) prepared in 40 mM borate buffer (TE). In total, 200 mM Tris-HCl was used as the LE and 138 V/cm as the EF. Inset images show the band formed during preconcentration.

Using these optimal ITP parameters, the preconcentration ratio of PB was determined. Since the intensities of the PB bands were observed using a fluorescence microscope, a calibration curve (Figure S2) was obtained with a limit of detection of 9.2 nM and this was utilized to quantify each concentrated PB band. Figure 3 shows the preconcentration ratios of PB from various initial concentrations (C_i_) under ITP. Since ITP concentrates the low concentrations of analytes, the preconcentration ratio increases as the initial concentration decreases. The preconcentration ratios (C_i_/C_f_) of PB range from a factor of 20–170,000 times and were obtained from initial PB concentrations, based on dilution calculations, of 10 nM to 100 fM respectively. This corresponds to detected concentrations between 200 and 17 nM, showing that previously undetectable amounts of PB were concentrated to detectable levels in our system. In a previous study, Alexa Fluor showed a million-fold preconcentration ratio using ITP coupled with capillary electrophoresis in a glass microfluidic chip [35]. In this study, we utilized a simply fabricated PDMS straight channel as a separate channel to perform 10^5^-fold preconcentration of PB. 

### 3.3. Preconcentrating LDH Using PB Conjugation

**Figure 4:**
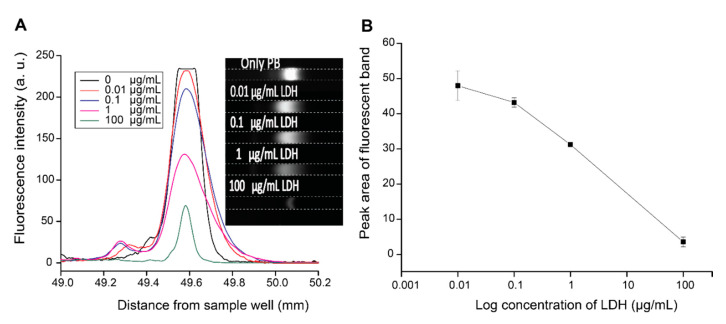
The indirect measurement of lactose dehydrogenase (LDH) using ITP. A series of LDH concentrations (0.01, 0.1, 1, and 100 µg/mL) were prepared and conjugated with PB. In total, 200 mM Tris-HCl for LE and 40 mM borate buffer for TE were used under 138 V/cm. (**A**) The intensity profile of the PB band during the ITP process with various LDH concentrations. (**B**) The peak areas of the PB bands are plotted with the log of LDH concentration.

To demonstrate the preconcentration of conjugated PB with a target analyte, we chose LDH to create the LDH-PB complex. LDH is a potential biomarker for different cancers [36] and many diseases, such as liver disease and myocardial infarction [37]. Although spectrometric methods have been reported to quantify LDH, LDH activity has often been measured by following either the oxidation of NADH with pyruvate or the reduction of NAD^+^, both of which involve several reaction steps [38,39]. In this work, by forming the PB-LDH complex with simple conjugation chemistry coupled with ITP, we successfully demonstrate how to quantify the amount of LDH using ITP. As shown in Figure 4A, the intensity of the fluorescent band decreased as the LDH concentration increased. By plotting the peak area of each LDH concentration, we obtained a calibration curve as a function of LDH concentrations, however camera saturation caused a plateau in the lowest concentration case. As seen in Figure 4B, the overall trend was an exponential decay with LDH concentration with a slight deviation at low concentrations due to the saturation. A previous study has shown the quenching effects that the protein itself can have on the fluorophore attached to it, using a series of Alexa Fluor dyes [40]. Therefore, the decrease of fluorescence intensity of PB may be due to the quenching effect of LDH that it has on PB; however, further study is needed. Using ITP under these optimized parameters, we were able to detect LDH concentrations in the range of 10 ng/mL to 100 µg/mL. Furthermore, to show the selectivity of this purposed method, we also performed LDH measurement with used HeLa cell media as a background matrix. Even under these conditions, increasing LDH concentration decreased PB band intensity (Figure S3). Thus, our purposed method of indirect measurement of LDH can be used in real/authentic samples obtained from cell cultures.

## 4. Conclusions

We performed an experimental parametric study focused on preconcentrating PB in a PDMS microfluidic chip by varying types of electrolyte, electric field density, measurement point, and electrolyte concentrations. An electrolyte system that was able to stack and separate PB was successfully characterized. The optimized parameters of the electrolyte system are as follows: 200 mM Tris-HCl at pH 8 as the LE, 40 mM borate buffer at pH 9 as the TE, 100 nM sodium acetate as a spacer, and 1% (*w*/*v*) PVP as the electroosmotic flow suppressor. A spacer is required to ensure that the hydrolyzed PB band does not interfere with the normal PB band. As we used a PDMS device to perform ITP, our approach achieved lower cost, less complexity, and shorter device-fabrication time than commonly used glass microfluidic chips. Detecting a trace amount of PB was demonstrated with a maximum preconcentration ratio of ≈ 1.7 × 10^5^ fold in 5 min. In addition, we introduced a direct method to detect LDH concentrations ranging from 10 ng/mL to 100 µg/mL that differs from conventional colorimetric methods by using an indirect measurement of PB. The ITP system proposed here has significant potential as a low-cost, simple, and effective tool for detecting LDH.

Previous work on detecting amino acids using CE reported that amine conjugated PB increases the measurement sensitivity [9,10,41,42]. By using ITP preconcentration, we measured the PB signal as low as 100 fM at given conditions of ITP. Coupling CZE with ITP can further enhance the measurement sensitivity of PB conjugated amines. In addition, both CZE and ITP require laborious sample preparation steps. Previously we demonstrated an automated CZE platform by using a programmable microfluidic platform (PMP) fabricated using soft lithography technique [27,43,44,45]. Since we characterized and optimized ITP conditions for PB in a PDMS microfluidic chip, this ITP system can be easily integrated with the PMP along with a laser-induced fluorescence (LIF) system [26,42,43,46] to realize a fully automated chemical analyzer with extreme sensitivity. The integrated platform will be highly field-deployable for environmental monitoring, disease diagnostics, and exobiology studies.

## Supplementary Materials

The following are available online at https://www.mdpi.com/2072-666X/11/11/951/s1, Figure S1: The impact of different EF densities on the accumulation of PB. LE and TE were 100 mM Tris-HCl and 40 mM borate buffer respectively, Table S1: Peak resolution at 30 mm and 40 mm measurement points under 138 V/cm EF.
