# Supplementary material for: Characterization and Optimization of Isotachophoresis Parameters for Pacific Blue Succinimidyl Ester Dye on a PDMS Microfluidic Chip
Source: Micromachines (Basel). 2020 Oct 22;11(11):951. doi: 10.3390/mi11110951 (PMC7690402; doi:10.3390/mi11110951)
Supplement: Supplementary file 1 [file micromachines-11-00951-s001.pdf]

micromachines
MDPI

Supplementary Information

Characterization and optimization of Pacific Blue Succinimidyl Ester dye for Isotachophoresis on a PDMS microfluidic chip

Himali Somaweera[1], Zachary Estlack[2], Jasmine Pramila Devadhasan[1], Jungtae Kim[3], and Jungkyu Kim[2,*]

[1]Department of Mechanical Engineering, Texas Tech University, Lubbock, TX 79409, USA

[2]Department of Mechanical Engineering, University of Utah, Salt Lake City, UT 84112, USA

[3]KIST-EUROPE, Saarbrucken, Germany

* Address correspondence to: Jungkyu (Jay) Kim

Department of Mechanical Engineering

University of Utah

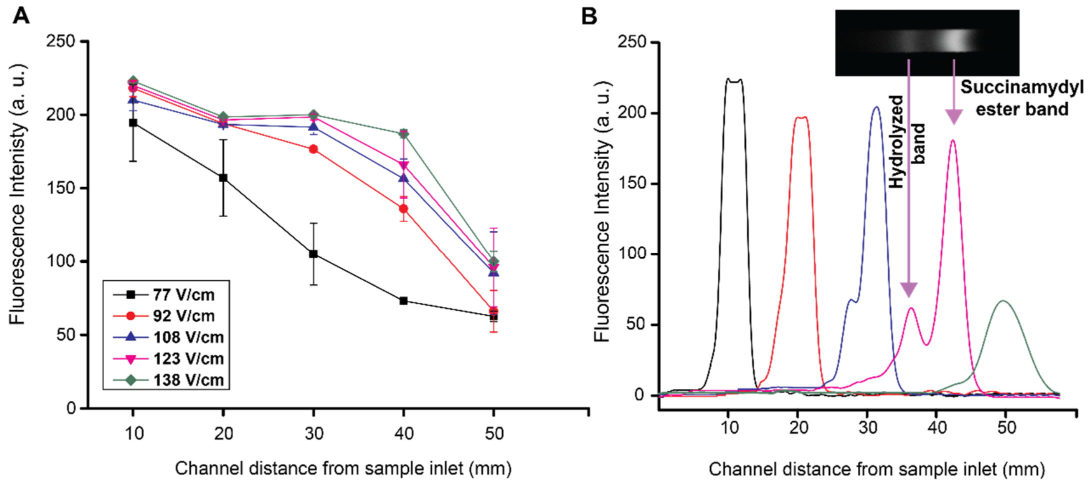

Figure S1. The impact of different EF densities on the accumulation of PB. LE and TE were 100 mM Tris-HCl and 40 mM borate buffer respectively, **(A)** The change in the average fluorescence intensity of the preconcentrated 100 pM PB from sample inlet to the outlet under 5 different electric field densities (77 V/cm, 92 V/cm, 108 V/cm, 123 V/cm, and 138 V/cm) (N=3). **(B)** Isotachophoragrams under five different measurement positions at 138 V/cm EF: black (10 mm), red (20 mm), blue (30 mm), magenta (40 mm), and olive (50 mm).

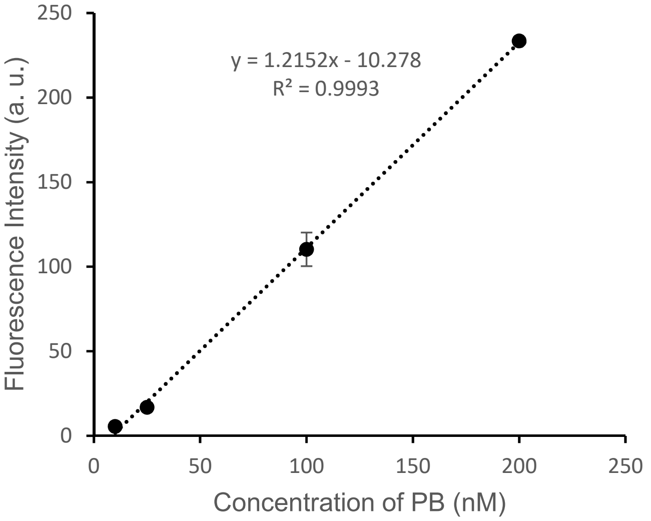

Figure S2. The calibration curve of four different PB concentrations(10 nM, 25 nM, 100 nM, and 200 nM) versus intensity without ITP. Statistical analysis gives a limit of detection of 9.2 nm.

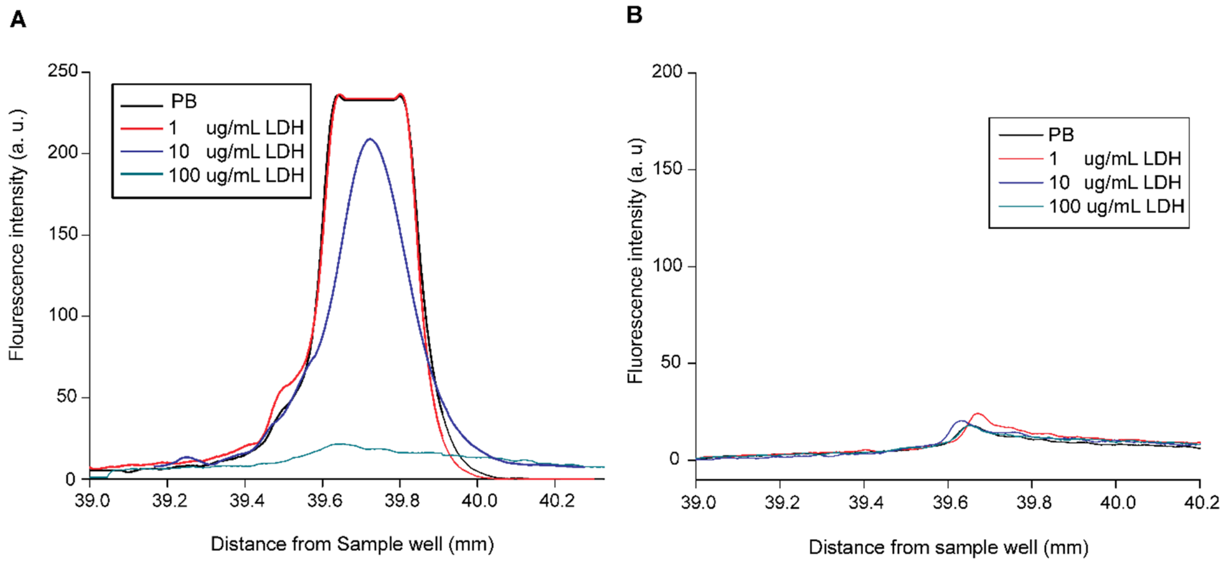

Figure S3 The indirect measurement of lactose dehydrogenase (LDH) using ITP when cell media was used as the matrix. A series of LDH concentrations (1 µg/mL, 10 µg/mL, and 100 µg/mL) were prepared and conjugated with PB. **(A)** The intensity profile of the PB band during the ITP process with various LDH concentrations and band formation. **(B)** The intensity profile of the second band that appeared much later than the main PB band.

Optimization of Electric field density and measurement location

During the selection of the electrolytes, an EF of 123 V/cm and the 30 mm measurement point was used. In ITP, as the EF is increased, the sample accumulation increases with various measurement locations [34]. Considering these facts, in order to get better separation and the highest accumulation, optimization of EF and measurement location is needed. After selecting electrolyte types, the electric field density and the measurement location were optimized. The lowest EF density was set at 77 V/cm (200 V) and then gradually increased. To avoid electrolysis at higher EFs, we chose 138 V/cm as the maximum EF density. As shown in Figure SI1A, 138 V/cm shows a minimal variation of intensity of the PB band between 20 mm and 40 mm. This result indicates that 138 V/cm provides a well-balanced PB band for minimizing intensity variation and was selected as the optimal EF. Furthermore, peak resolution was evaluated at five different measurement points using the two PB peaks as an indicator to choose the proper measurement point. Figure SI1B shows the intensity profiles at five different measurement points downstream from the sample inlet under 138 V/cm EF. By calculating and comparing the peak resolution presented in supplemental information, we found that the maximum resolution is at 40 mm from the TE well (Figure SI2 and Table SI1) and was used for all further experiments.

Optimization of the LE and the TE concentrations

In general, the conductivity between the LE and the TE increases with the increase of buffer concentrations, resulting in a high electrophoretic flux of samples from the TE zone to the stacked sample zone during ITP [1]. With all previously optimized ITP parameters, further optimization is needed by determining proper electrolyte concentrations of both LE and TE. Figure S1A shows the intensity profiles of the PB band from five different Tris-HCl concentrations (between 100 mM and 250 mM) at the 40 mm measurement point. Although the highest accumulation of PB was observed with 250 mM Tris-HCl, the resolution was too poor to distinguish two bands. By comparing the profiles of the PB bands, 200 mM Tris-HCl shows less deviation than the concentrations (Figure SI2).

To determine the optimal TE concentration for PB preconcentration, 40mM, 60mM, and 80mM of borate buffers at pH 9 were evaluated under 200 mM Tris-HCl and 138 V/cm EF (Figure S1B). 40 mM borate buffer showed a higher accumulation than that of 80 mM with a similar band resolution. Although 60 mM borate buffer showed the highest accumulation of PB, the band resolution was poor compared to those of the other two borate concentrations. From these characterizations, 40 mM borate buffer for TE with 200mM Tris-HCl for LE are the best buffer combination for PB preconcentration using ITP with a PDMS microfluidic chip.

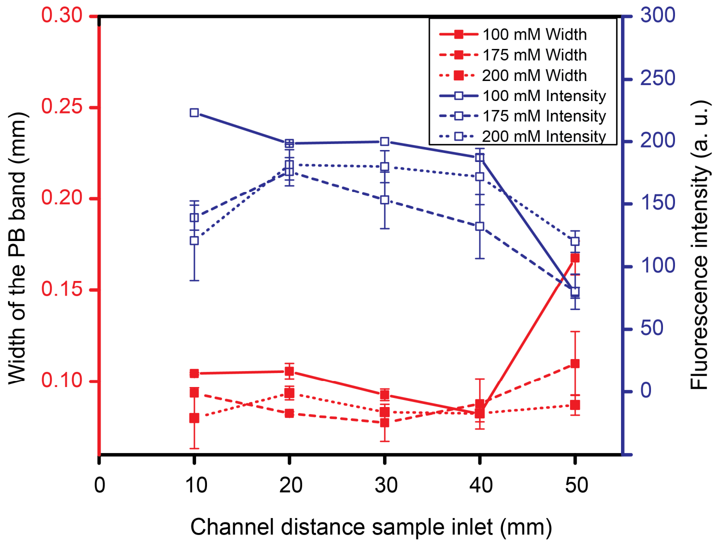

Figure S4. Change in the width of the PB band and the average intensities for 100 mM, 175 mM, and 200 mM LE at five measurement points downstream of the sample inlet. 40 mM tetraborate was used as TE, and 138 V/cm electric field was applied.

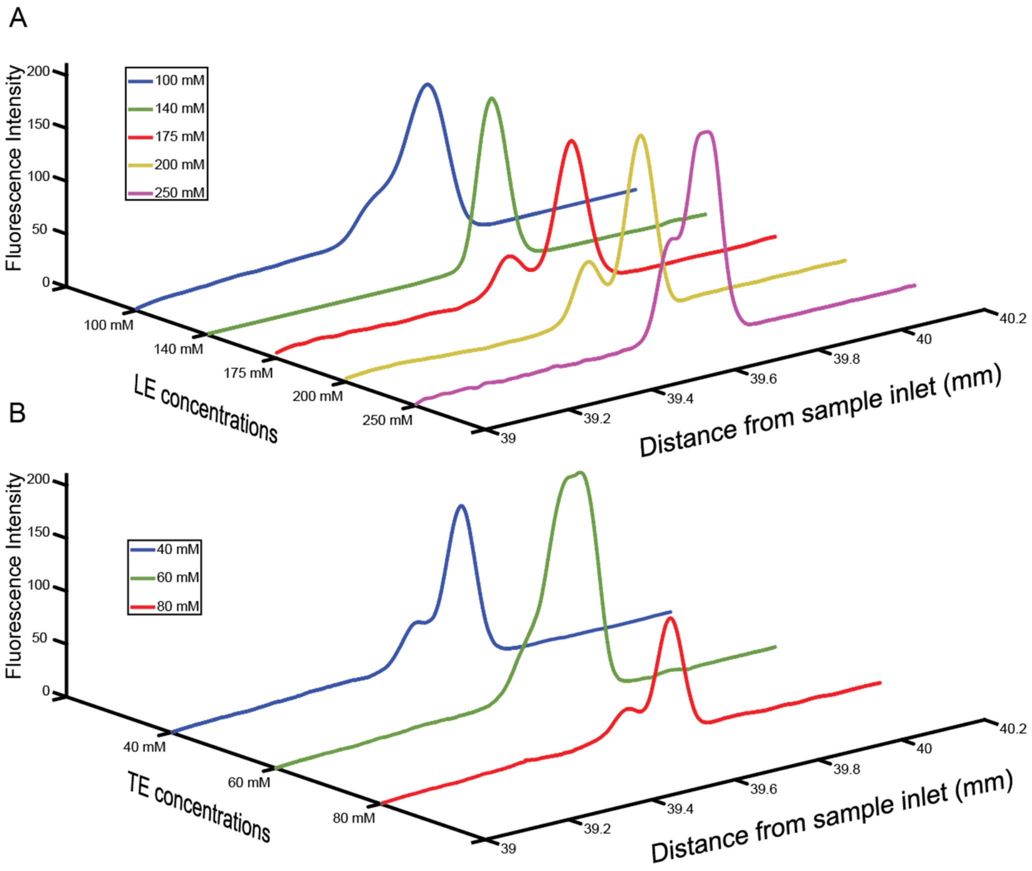

Figure S5. The effect of electrolyte concentrations on 100 pM PB preconcentration. The intensity profiles of the PB bands under various concentrations of LE (pH 8 Tris-HCl) and TE (pH 9 borate buffer) were obtained at 40 mm downstream from the sample inlet. **(A)** Intensity profiles from five different concentrations of Tris-HCl (100 mM, 140 mM, 175 mM, 200 mM, and 250 mM) with 40 mM borate buffer under 138 V/cm. **(B)** Intensity profiles from three different concentrations of borate buffer (40 mM, 60 mM, and 80 mM) with 200 mM Tris-HCl under 138 V/cm.

Calculation of Resolution:

The resolution (R) was calculated using the distance between two peaks and the full width at half maximum (FWHM). The standard deviations (σ) are calculated for FWHM. The following equation was used to calculate the resolution [2], where t_1, t_2 are retention time for each peak, and $W_{0.5h1}$ and $W_{0.5h2}$ are FWHM of each peak.

$$R = 1.18 \times \left[\frac{t_2 - t_1}{W_{0.5h_1} + W_{0.5h_2}}\right]$$

Table SI1. Peak resolution at 30 mm and 40 mm measurement points under 138 V/cm EF.

Measurement point (mm)	Resolution (R)
30	0.329
40	1.014

Table SI2. Optimized buffer conditions and their concentrations

Type	Electrolyte/chemical	Concentration
Leading electrolyte	Tris-HCL at pH 8	200 mM
Trailing electrolyte	Sodium tetraborate at pH 9	40 mM
EOF suppressor	Polyvinylpyrrolidone	1 % (w/v)

References:

1. Jung, B.; Bharadwaj, R.; Santiago, J.G. On-chip millionfold sample stacking using transient isotachophoresis. *Anal. Chem.* **2006**, *78*, 2319-2327, doi:10.1021/ac051659w.
2. Luckey, J.A.; Norris, T.B.; Smith, L.M. Analysis of Resolution in DNA Sequencing by Capillary Gel-Electrophoresis. *J Phys Chem* **1993**, *97*, 3067-3075, doi:DOI 10.1021/j100114a038.
